# Innovation in a backwater: The Harpurhey Resettlement Team and the mental health services of North Manchester, 1982–1987

**DOI:** 10.1016/j.healthplace.2009.02.008

**Published:** 2009-09

**Authors:** Val Harrington

**Affiliations:** Personal Social Services Research Unit, Dover St. Building, University of Manchester, Oxford Road, Manchester M13 9PL, UK

**Keywords:** Mental health services, History, Innovation

## Abstract

This paper explores the circumstances around the setting up of the Harpurhey Resettlement Team, an innovative project which, in the late 1980s, resettled around 20 long-stay patients from Springfield Hospital in North Manchester into ordinary tenancies within the same neighbourhood. It argues that Springfield's position as a marginalised and neglected institution produced the conditions for such innovation; while the particular and unexpected convergence of national policies, local structures and institutional politics created space for a process of change which, in both form and outcome, could not have occurred in the more regulated psychiatric environments elsewhere in Manchester.

## Introduction

So I got to know … I can’t remember if it was two people or one person, but the person I remember is Winifred,^1^ who was a woman living in Springfield, on a ward in Springfield, and as part of the ‘Getting to Know You’ process we had to be with them in the hospital, had to be with them outside and then after that we had to read the notes. But it was [pause] it made the planning process into a very personal thing, which was .. which was wonderful. You know, it wasn’t about just some group of people over there, you became attached to this particular person and wanted to make the plan work for them.

(Interview 1: Research Assistant, *Getting to Know You Project*)

The above quotation provides an unusual introduction to a paper which is essentially about service development. Firstly, the speaker—a young Cambridge graduate, working initially as a volunteer and later on a temporary research contract funded by the Manpower Services Commission—is not a figure one would normally associate with strategic service planning. Secondly, neither his language nor tone is typical of someone engaged in the purportedly rational and objective activity of service design. And thirdly, establishing a deeply personal relationship with an elderly woman, incarcerated on a psychiatric backward for the past 40 years, is hardly the starting point most planners would choose for developing new ways of delivering mental health services to the local community. But then, the service around which this paper is based was highly unusual—and, indeed, in terms of both origin and intent, represented a fundamental challenge to the prevailing models of both mental illness and mental illness services.

The service in question was the Harpurhey Resettlement Team which, in the late 1980s, resettled between 20 and 25 long-stay patients from Springfield Hospital in North Manchester. The project differed from traditional resettlement programmes in a number of key ways. Firstly, the accommodation took the form of ordinary tenancies—individually rented flats and houses as opposed to the more usual pattern of hostels, group homes and sheltered housing schemes. Secondly, the whole project was built around the notion of neighbourhood. The entire cohort moved to the same geographical district, a small, well-established neighbourhood of approximately one square mile in area, and from the beginning the focus was as much on developing links with the local community as on clinical practice—something which was underlined both by the presence of an experienced community development worker in the team and by the fact that a significant proportion of support workers were appointed on the basis of their connections with the area, rather than any direct mental health experience. Thirdly, despite this broad range of personnel, skills and backgrounds, the team structure was explicitly non-hierarchical, with no team leader, decision-making by consensus, skill-sharing and, in the early stages, broad parity between the support workers in terms of both pay and job descriptions. Given that half the support workers were qualified psychiatric nurses employed by the health authority, and half were social services workers who were mainly unqualified, local people, this latter feature was particularly unusual—as, indeed was the fact that the project was jointly managed by health and social services. Finally, the team was closely involved with radical mental health issues and organisations, and played a part in the setting up of the independent and highly vocal *North Manchester Users’ Group*.^2^

The focus of this paper will be on the setting up of the service: the context, both national and local, within which it developed; the drivers and mediators of change; and the mechanisms and processes involved. Clearly, the actual number of patients resettled by the team represents only a small proportion of the hospital's several hundred long-stay population. In symbolic terms, however, the project was hugely significant, occupying a central position in the massive political upheavals which accompanied the transfer of services away from the old mental hospital into new settings. As such, the case study provides a fascinating window through which to explore the dynamics of change and, more specifically, to trace the interplay between the national and the local: not only how national policies were implemented and interpreted on the ground but also how broader social and political themes were played out within the context of local service development. Within this, the notion of place plays a central role: firstly, in relation to the actual service model; secondly, in terms of Springfield itself and the ways in which its very particular history and geography created the conditions for non-mainstream forms of innovation; and thirdly, echoing Spandler's notion of ‘convergent space’, as a particular ‘place in time’, in which a unique—and in this instance often contradictory—combination of political forces and social actors came together in one ‘specific, local convergent site’ (Spandler, 2006, p. 23).

My sources are predominantly oral, drawn from interviews with practitioners and service providers which have been conducted over the past 3 years as part of my PhD, a broader post-war history of mental health services in Manchester and Salford (Harrington, 2008). Recruited largely through the ‘snowballing’ technique (Crossley, 2006), my 22 North Manchester informants represent a range of perspectives and occupational backgrounds: from key figures within the services to frontline workers; from the drivers of innovation to those who were required to adapt and implement the changes within their day-to-day practice; and from opponents as well as advocates of change. What is clearly missing is the voice of service users. This is due to the constraints of both time and space and reflects how, with a primary focus on service dynamics and organisational issues, staff rather than patients were the natural target group. The oral testimonies are augmented by documentary evidence in the form of reports, minutes and service materials.

## The backdrop to the services

The story begins in 1982, towards the end of Margaret Thatcher's first term in office and a period of deep political and social turmoil. The public services were, in effect, being dismantled, as the welfare state was transformed into a ‘mixed economy of welfare’, characterised, according to Rogers and Pilgrim (2001), by the triple themes of ‘privatisation, marketisation and managerialism’. The other side of Thatcherism was the huge wave of opposition that her policies fuelled and the new generation of left wing activism they spawned. At a local level this opposition took a particularly interesting form, with the springing up of loose networks of likeminded people, mobilising around specific issues and projects. A significant proportion involved the reframing of traditional health or welfare issues within a broader, left wing agenda, and the drawing in of actors and resources from a range of backgrounds and perspectives—influencing services in ways which had not been predicted and creating and fostering strong links across various sectors and levels of service. These links were to prove pivotal both in this story, and more generally in the increasingly complex and fluid world of the late twentieth century British health and welfare services.

### The mental health context: deinstitutionalisation and mental health activism

Speaking personally, I felt that there were three currents, three strands that were going. One was the ‘Survivors Speak Out’^3^ stuff, so it's actually service users’ autonomous voice work that was coming…the second one was very much our strand, which was the resettlement programme, because it wasn’t just us, it was happening across the UK…And then the third one is the…legacy of people like Laing and so on, Cooper,^4^ so people who had set up alternative, counter-therapy type.. therapeutic communities… and it was that melange of the three that I felt..heady days.

(Interview 2: Nurse on the Harpurhey Resettlement Team)

Within this broader context of economic and public service reform the mental health services were facing their own particular pressures, brought on by an accelerating programme of deinstitutionalisation. The run down of the large mental hospitals had been firmly on the agenda since Enoch Powell's famous ‘water tower’ speech of 1961,^5^ but progress over the subsequent two decades had been slow and gradual. In 1975, for example, the Government White Paper, *Better Services for the Mentally Ill*, had highlighted how, although mental hospital populations were falling, levels had not yet halved and no institution had closed. Its publication represented an important landmark in mental health policy and an unequivocal endorsement of district-based psychiatric services, supported by a range of facilities within the community. In the increasingly harsh economic climate of the late 1970s, however, both Regional Health Authorities and Local Authority Social Services Departments were generally slow to respond—a position which pertained until 1984, when a government directive was sent out to the Regional Health Authorities, requiring them to produce definite plans for the run-down and closure of all large mental hospitals in their region (DHSS, 1975; Jones, 1993; Busfield, 1986; MIND, 1983; NWRHA, 1985). This heralded a flurry of activity throughout the country: between 1985 and March 1993 the number of mental hospital beds fell from around 60,000 to just over 20,000; 31 institutions closed; and, of those that remained, most had firm plans to shut before the end of the century (Davidge et al., 1993). Community care—one of the cornerstones of the 1959 Mental Health Act—was finally to become a reality and the mental health landscape was set to change almost beyond recognition.

At the same time, a very different kind of mental health movement was also gaining momentum. In what arguably constituted the second wave of ‘anti-psychiatry’, this movement paralleled, and often overlapped with, the forms of political opposition already discussed. Again, rather than one distinct entity it consisted of a loose network of individuals and groups, united in their general opposition to mainstream psychiatry but, in terms of background and philosophy ‘a very diverse, fluid, frequently contradictory counter-culture’ (Greenwood, 2004, p. 1). Within this, a number of key themes can be identified. The first centred around radical alternatives to service provision, and were epitomised by the Marxist-inspired services in Trieste in Northern Italy (Crossley, 2006; Jones, 1993; De Leonardis et al., 1986); the second concerned broader issues of civil liberties and human rights; and the third was the growth, in the second half of the 1980s, of the service user movement (Crossley, 2006; Rogers and Pilgrim, 1991; Campbell, 2005). The latter's uneven emergence into what was already a fluid and turbulent field disturbed and eventually transformed the landscape of mental health activism at a time when the mainstream mental health services were also undergoing tremendous change. The story of the Harpurhey Resettlement Team is, to some extent, the story of how these two, traditionally opposing forces both interacted and collided with each other in the context of Thatcher's Britain and a marginalised and previously neglected mental health service.

### North Manchester and its mental health services

North Manchester always felt the poor relation … Nobody really cared. I don’t say nobody cared, but it felt a bit like that. It was more important that the teaching hospital bit survived. You didn’t have to worry about crumbling old buildings up here.

(Interview 3: Member of North Manchester DHA)

The thing about Springfield Hospital…was that it was such a backwater of a place that it had no pretensions or illusions…it was the arse-hole of the universe.

(Interview 2: Nurse on the Harpurhey Resettlement Team)

**Fig. 1:**
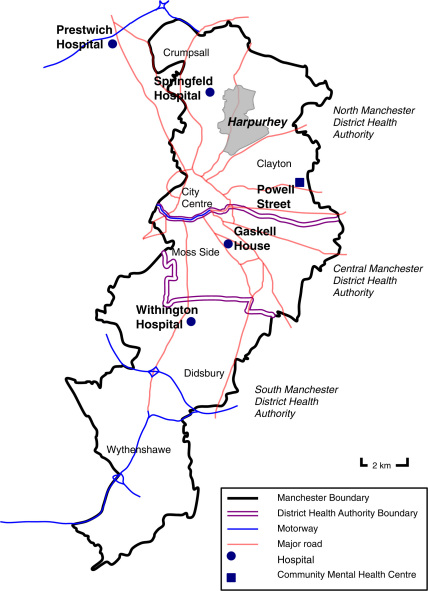
Map of Manchester showing district health authority boundaries, main psychiatric hospitals and location of North Manchester community mental health facilities.

Of Manchester's three district health authorities—South, Central and North—North Manchester, whose population in 1987 stood at just under 143,000, accounted for some of the highest indices of deprivation and health inequalities in the city. Extending northwards and eastwards from the city centre, eight of its eleven wards were within inner city areas and much of its landscape was a testament to post-industrial Britain. Many of its traditional industries had closed down, leaving in their wake the highest level of airborne pollution in the country and disturbing levels of unemployment and hardship. This translated into a depressing collection of economic and public health statistics: data compiled from the 1981 census, for example, placed the majority of North Manchester's wards near the bottom of Greater Manchester's deprivation and health rankings (Townsend, 1988); in 1987 its standard mortality ratio of 134 was the highest in the region; and the authority's illegitimacy and low birth weight rates in 1988 were almost twice the average for England and Wales (North Manchester DHA, 1989). Harpurhey was no exception. Situated just over two miles north of the city centre, with a population in 1991 of around 17,000, it was a traditional white, working-class district. In 2004 it gained the dubious honour of being named the most deprived neighbourhood in England, with the poorest quality of life in the country (Tapp, 2004) (Fig. 1).

In 1982, when the story starts, North Manchester's mental health services were centred around Springfield Hospital,^6^ one of Manchester's two large mental hospitals. Opened in 1858, Springfield was unusual in terms of both its history and geography, which set it apart both from the region's purpose-built asylums and the district general psychiatric units described in the introduction to this theme section (Philo and Pickstone, 2009). It had started life as Crumpsall Workhouse, built to relieve pressure on, and eventually replace, existing facilities in the centre of Manchester. Over the subsequent 60 years it became increasingly populated by the elderly and ‘adults of unsound mind’ and under the 1913 Mental Treatment Act was officially designated as an institution for housing the feeble-minded. It still remained under the jurisdiction of the Poor Law, however, as one of several specialised institutions run by the Manchester Poor Law Union—indeed, by the 1920s, the Manchester Board, which had merged with the Boards for South Manchester and Prestwich in 1915, was running a children's hospital (Booth Hall), an epileptic colony (Langho) and a facility for overnight accommodation, in addition to its general poor law accommodation. It also boasted two major hospitals, Withington and Crumpsall, the latter having been built as the infirmary for the Crumpsall Workhouse. Under the reforms of the 1929 Local Government Act responsibility for Springfield (at that time known as the Crumpsall Institute) was transferred from the Board of Guardians to Manchester's Public Assistance Committee (Greenwood, 1998), while the Crumpsall Infirmary was taken over by the Health Committee.

This separation continued during the first 25 years of the NHS. Under the 1946 NHS Act, workhouse accommodation for the mentally ill and subnormal—which generally comprised a few wards in the ex-municipal hospitals—was transferred to the health service. In most towns, such wards now came under the remit of a local Hospital Management Committee. HMCs were responsible for administering the ex-municipal and former charity hospitals in each large town or city-district; significantly, they had no formal connections with the large asylums which, often distant from population centres and very much ‘worlds in themselves’, were given their own separate HMCs. Unusually, and despite its urban location and proximity to Crumpsall Hospital, Springfield was treated as an asylum: thus, rather than joining with Crumpsall and other hospitals in North Manchester, it was grouped with another former poor law institution, the Swinton Home for mentally subnormal children, to form its own HMC. Springfield's workhouse origins, however, meant that it remained marginal to the constellation of major asylums inherited by the Manchester Regional Hospital Board from the Lancashire Asylums Board—whilst also being separated under the NHS from the institution with which it shared a site. Springfield was indeed peculiar, and it was to remain marginal in several ways.

Firstly, although in the early 1970s Crumpsall and Springfield eventually amalgamated to form North Manchester General Hospital, Springfield was always the outsider. Despite their shared history and close physical proximity, and despite the general push during that period to move psychiatric services out of the asylum and into district general units, Springfield never succeeded in integrating with the rest of the hospital:

Even within the hospital hierarchy, it was Crumpsall and Springfield and they had been two separate hospitals. They were just forced together-they’d been in different hospital groups…and I mean ridiculous separation such as that if somebody had an accident or took ill or something on the Springfield site nobody wanted to go across to deal with them, you know, from A&E or surgery or medicine, etc. Nothing to do with them, sort of thing.

(Interview 4: Member of North Manchester DHA)

There was [pause] a precipice is perhaps too strong a word to use, but the atmosphere was very.. there was a very marked contrast between Springfield and Crumpsall.. the acute hospital, even though they were now part of the same organisation, and I guess in some ways it was like stepping back into time.

(Interview 5: Member of North Manchester DHA)

The brick wall which had originally separated the two institutions had long since been removed (Greenwood, 1998) but the cultural brick wall clearly still remained—with Springfield definitely on the wrong side.

Secondly, Springfield was the least powerful of the North West Regional Health Authority's four large mental hospitals. This was partly due to its late entry into the mental hospital system, but was also a function of its size and geography. Compared to the neighbouring Prestwich asylum, for example, Springfield was tiny: in the early post war period, when bed numbers were at their highest, Prestwich's population peaked at over 3000 while Springfield's barely rose above 600; and by the early 1980s, when Prestwich still had in excess of 1000 patients, Springfield's population had fallen to a few hundred (Hopton, 1999; Danson, 1985; NWRHA, 1985). In contrast to the purpose-built asylums it was also small in terms of acreage: while Prestwich was surrounded by extensive parkland, the Springfield site was almost completely built up. The issue of land availability assumed a whole new significance in the context of the regional health authority's plans for psychiatric services during the 1970s and 1980s. Integral to these was the setting up of a range of specialist facilities to complement the region's district general units (Harrington, 2004)—a development which, according to Hugh Freeman, was only possible because the large mental hospitals were able to provide both the land and the infrastructure to support them:

It was only there that space and supporting facilities could be found to develop the new sub-specialties of psycho-geriatrics, rehabilitation, drug abuse and forensic psychiatry, which became the most highly developed in the world…Whether it would have been possible without the presence of the mental hospitals is an interesting question.

(Freeman, 1999, p. 7)

By 1984 Prestwich had 90 new specialist beds—a 44 bedded forensic unit, an adolescent ward and two centres for the treatment of alcohol and drug dependency (NWRHA, 1985)—while Springfield had been completely sidelined.

Thirdly, as a predominantly long-stay institution Springfield was similarly sidelined within Manchester's wider psychiatric community. Manchester psychiatry was dominated by the University Department of Psychiatry and its relatively new academic units at Gaskell House in Central Manchester and Withington Hospital in South. These espoused a model of acute, district general psychiatry within a fiercely competitive and academic environment (Kessel, 1973; Harrington, 2008)—a far cry from the back-wards of Springfield. The cultural divide between Springfield and its sister units in Central and South Manchester was underlined by Springfield's lack of teaching status, which further compounded the vicious circle of isolation and mutual suspicion.

Finally, this cultural and political divide was not confined to psychiatry. North Manchester was the only health authority in Manchester not to have a teaching hospital—a factor which was not only played out in the health politics of the city but which also helped to define how the district saw itself:

North Manchester had.. kind of had this chip on its shoulders. I remember when I first went to, got into association with the Area through the CHC, I thought it was astonishing really, this, ‘Oh, we never get anything, we’re the poor relations, we haven’t got a medical school and so on’.

(Interview 5: Member of North Manchester DHA)

Springfield's already marginalised status was thus compounded by being part of a health authority which, in terms of power, prestige, resources—and, indeed, self-image—was at a huge disadvantage compared with its near neighbours.

This marginalisation translated into some appalling conditions at ground level. Built over a century earlier and for a very different purpose, much of the Springfield accommodation was inappropriate and in a poor state of repair. More disturbing than the physical conditions, however, were the attitudes and practices of a dominant section of the workforce which were more akin to Springfield's workhouse origins than to a modern psychiatric hospital:

The place was such an utter disgrace. I mean so terrible, the environment and the attitudes… the conditions were horrific… as well as the appalling physical conditions that there were, people not having their own clothes, nowhere to keep any possessions, and all this sort of stuff.

(Interview 4: Member of North Manchester DHA)

The extent of the problem was highlighted in a series of damning external reports published in the early 1980s, including one from the Hospital Advisory Service which described Springfield as ‘one of the worst hospital sites they had visited in the country’ (Social Services Inspectorate, 1986, p. 34).

Given this situation, it is perhaps surprising that North Manchester became a site of innovation. I would argue, however, that it was its very status as a backwater which created some of the conditions for innovation. Firstly, away from the scrutiny and influence of Manchester's medical and psychiatric fraternities there was more freedom to experiment outside conventional channels; and secondly, Springfield was politically very weak. This meant that although there was huge internal resistance to change, this resistance was ultimately feeble since it lacked support from outside. Operating almost in a political vacuum, Springfield was clearly a prime site for revolution!

## The who and the how of change

### The drivers of change

She was very unusual, in that she just wasn’t concerned with her own professional power there. I mean she tried to use it to make something happen but she was equally at home with .. you know, she basically wanted other people who were thinking in the same way that she was, rather than anything else. And she operated in a very different way from most doctors that you might ever come across, and that went on in her personal life as well, and her personal life and her kind of public life were not particularly separated, and so once you became part of that kind of … set of people you were part of that set of people. So it was very, it's part of a radical milieu that was around at that time.

(Interview 1: Research Assistant, *Getting to Know You Project*)

Interestingly—though, given the politics of Springfield, not altogether unsurprisingly—the initial drivers of change came from outside the institution. They also had a non-psychiatric background: Drs. Joyce Leeson and Judith Gray were in North Manchester's Department of Community Medicine, which after the NHS re-organisation of 1974 had inherited many of the local functions of the city's Public Health department. Leeson and Gray joined the newly autonomous health authority in 1982. Both were committed feminists and left wing radicals, who spent their professional lives explicitly working towards a more democratic health service through greater participation, collaboration and involvement of patients, local communities and staff at all levels (Leeson and Gray, 1978; Leeson, 1991).

Joyce Leeson was North Manchester's District Medical Officer and extremely well respected by the chair of the district health authority (DHA), who had encouraged her to apply for the post.^7^ As the senior local figure in Community Medicine and a member of the DHA, her main role in this story is that of supporter and enabler—backed up by a significant degree of power and influence: as one interviewee observed, ‘Joyce certainly was a terribly powerful woman. Terribly powerful’ (Interview 6: Member of the *Getting to Know You* Core Planning Group). This influential position at the strategic level was complemented on the ground by the less conventional, but equally formidable force of her newly appointed Specialist in Community Medicine, Judith Gray. Judith was, by all accounts, a strongly charismatic figure, variously described in my interviews as ‘an inspirational sort of person’ ‘a very charismatic public health doctor’ ‘a huge maverick’ and a ‘rather wonderful woman’. Despite holding a senior position in the medical hierarchy she was, in essence, a grassroots activist who was deeply connected, in a myriad of ways, to the oppositional movements and networks described above. It was this grassroots approach which characterised her response when, in June 1982, she was invited by the DHA to review plans for a proposed psychiatric day centre.^8^ Two months later her remit was considerably broadened when she volunteered to coordinate a service development strategy exercise focussing this time on Springfield's long-stay population.

### The mechanics of change: Getting to Know You

The whole thing was about giving people back their dignity and giving them a life back.

(Interview 7: Nurse/Nurse Manager at Springfield Hospital)

The vehicle of change was *Getting to Know You*: a project which, in contrast to more conventional approaches to service planning, was designed to involve and engage as many staff—and, indeed patients—as possible. Explicitly built around the principles of normalisation (Wolfensberger, 1972; Brown and Smith, 1992) and based on a Canadian scheme of the same name (Brost and Johnson, 1982), its ‘bottom-up’ rather than ‘top-down’ approach would, it was claimed, create a very different type of service: one which was defined and shaped by the needs of real users rather than the goals and constraints of the organisation.

The project was in two stages. The initial phase took the form of a detailed individual assessment exercise. Around 20 volunteers, a mixture of managers and practitioners, took part—although the method of recruitment, a series of meetings ‘almost like a moving road show selling the opportunity to discuss and change present services’ (Thomas and Rose, 1986, p. 4), and the nature of the exercise itself created a much higher level of visibility within the hospital than these numbers might suggest. The assessments, designed to ‘strip staff of their ‘professional’ assumptions and allow them to feel again the raw experience of the lives of those who use our service’ (Thomas and Rose, 1986, p. 4), involved spending many hours ‘getting to know’ an individual patient; slowly building up a detailed picture, both of the them as people—as opposed to clinical entities—and of the pattern and quality of their daily lives. Once completed, the assessments fed directly into the service design phase, to which representatives from social services and MIND were also invited. This culminated in the creation of a set of service design principles, which, it was proposed, should underpin any future resettlement service—and which was eventually to become the bedrock of the Harpurhey Team.^9^ In line with its normalisation roots, the emphasis was on enabling individuals not only to live in their local community but to experience an ordinary lifestyle, with genuine access to local facilities and activities and meaningful contact with local people. While considerable attention was thus paid to the identification and use of community resources there was no mention of specialist services and facilities nor of the medical aspects of care.

Unsurprisingly, given the impoverished lives of most hospital residents, the very personal nature of the assessment process had a profound impact on many of the participants, evoking anger and distress and causing them to re-appraise both their patients and their professional practice (Thomas and Rose, 1986). Indeed, a striking feature—and an undoubted strength—of the *Getting to Know You* project was the way in which its radicalism was balanced by a deep humanitarianism. This meant that it had a very broad appeal, bringing together two very different groups with very different world views. So, while the agenda for some was strongly political, confronting issues of power, authority and control, for others the issue was more immediate and practical:

This wasn’t just about a support service to twenty people … the whole process was one of transferring resources into the community so that they became community resources, not just resources for those twenty people … That de-institutionalisation is not just going to be a micro-project to set up nice little new services, it's about the whole processes of institutional practice… it's about the power of professionals, it's about… how much power the community has, and is reflective of an era …and the politics that challenged institutional authority.

(Interview 2: Nurse on the Harpurhey Resettlement Team)  

The ‘Getting to Know You’ stuff was about just that, we can build a service for people if you get to know ‘em. Get to know what they want out of life, what services they’ve had, what kind of experiences they’ve had, what they want, what they can do, what they can’t do, what their strengths are, their assets, you know, all that kind of stuff, and it was a new way of working…It was a new, good way of working … I thought, bloody hell, this is a real refreshing way of ..working with people and just more human way…so I sort of bought into it. … But I didn’t have the idea that ..let's bring down the traditional way of doing something, I just thought, you know, let's see if there's a better way of doing it. I wasn’t openly that radical or that trailblazing or carrying flags or anything like that, I just wanted to do a good job in a different way, I think.

(Interview 8: Nurse on the Harpurhey Resettlement Team)

This unlikely alliance of mental health activists and more conventional, practice-oriented professionals created a powerful force, uniting senior managers, staff at ground level and a range of actors from outside the institution, in what Thomas and Rose (1986, p. 18) refer to as ‘an atmosphere of change’.

Support was, however, far from universal. The psychiatrists had already demonstrated their disapproval of the project by choosing not to participate in the original working group. When the final report was put out for consultation in September 1984 this disapproval turned into massive opposition. According to many supporters of the scheme this was largely about professional power and status:

We were dismantling their empire, it's a simple as that. Their empire was this catchment in-patient population that they could do what they wanted to. They didn’t have a voice, they didn’t have a say in their treatment, they didn’t have much of a say in their care and they were overmedicated in many cases. And we were saying to the consultants ‘Actually we’re going to close this place down and you won’t have a job’.

(Interview 7: Nurse/Nurse Manager at Springfield Hospital)

What was under threat for the psychiatrists was not only their traditional constituencies—the several hundred in-patients who constituted the bulk of North Manchester's mental health population—but the relationships which structured those constituencies and which, more importantly, defined and confirmed both the psychiatrists’ professional identity and their position within the organisation. Given their already marginalised position, it is thus not surprising that Springfield's psychiatrists felt especially vulnerable and outraged—particularly since the threat was coming from a number of directions, both inside and outside the hospital, and was being successfully spearheaded by a female public health doctor who, in traditional terms, was even lower on the pecking order than themselves.

These negative responses were not driven by self-interest alone, however. For many—psychiatrists and nursing staff alike—*Getting to Know You* represented a betrayal, not only of their professional values and practices, but, more importantly, of the patients themselves. There were serious doubts about both the ability of many long-stay patients to survive in the outside world and the ethics of moving people out of what had effectively become their home:

We got an awful lot of stick…from other, you know, people…saying, ‘Poor Freddy, look at, how can you be exposing him to that kind of risk? How can you… he's warm, he's safe, he's this, that, and the other here, this is his home, this is his life. What right have you got?’

(Interview 8: Nurse on the Harpurhey Resettlement Team)

With such polarised views, the debate became increasingly acrimonious—something which is reflected in the strong emotions and highly charged comments evoked during some of my interviews, conducted over 20 years after these events. Although often couched in quite personal and derogatory terms—incompetent, retrogressive psychiatrists versus naïve and dangerous subversives—the problems were, however, ultimately more structural and conceptual in nature; the product of a power struggle between two very different cultures, fuelled on one side by anger and hope, and on the other by a deep sense of threat and betrayal.

### The mediators of change: North Manchester DHA, the dowry system and general management

Let's be clear, if that dowry system hadn’t appeared we would now not be talking, we’d just be talking about some nice piece of paper that some of us produced at some point, because without that we’d never have had the money to make it happen.

(Interview 1: Research Assistant, *Getting to Know You Project*)  

Griffiths has got a lot to be proud of … none of these things that we’re now talking about, even given the sort of promising starts, could have happened without general management.

(Interview 9: District Psychologist)

At this stage in the story it is tempting to view the eventual launch of the Harpurhey Resettlement Team as a political victory for Judith Gray and the direct outcome of the *Getting To Know You* project. And indeed, during the early stages of my research this is how I understood it. However, although *Getting to Know You* played a pivotal role in the creation of Thomas and Rose's ‘atmosphere for change’, it was in itself an insufficient condition for that change. What was needed was a supportive infrastructure within which the reformers’ ideas and energy could be translated into concrete action. This support came from three separate sources. The first was the district health authority which, as the body ultimately responsible for the quality of North Manchester's health services, had the power to drive through the changes. In the wake of a series of adverse inspections it also had the incentive:

The health authority was responsible for those services that were provided, and because they were seen as unacceptable ..as I said, if you’ve had a number of external reports which raised questions about what's happening, you cannot ignore that. It's at your peril if you do.

(Interview 5: Member of North Manchester DHA)

Perhaps less obvious are the reasons why the DHA chose to listen to a public health doctor rather than consulting more conventional sources of psychiatric wisdom—particularly given the proximity of Manchester University's Department of Psychiatry. First was the figure of Joyce Leeson, whose influential position enabled her to mediate between Judith and the board members, particularly the chair. Secondly, the timing was favourable. Judith joined North Manchester at a time when its services were being looked at afresh by a newly appointed health authority, eager to make its mark.^10^ Not yet ground down by the inevitable setbacks and pressures of NHS politics, the ‘infant’ committee was more likely to view innovative ideas with enthusiastic optimism rather than cynical defensiveness. Ultimately, however, it was, in the words of one of its members, about:

the kind of authority it was … the collection of people who were there … People who would ask questions and not accept the status quo.

(Interview 5: Member of North Manchester DHA)

This was in part a product of the character and leadership style of the chair, Joe Moore (Jones and Pickstone, 2008), and in part a consequence of the particular mix of committee members. Unlike its counterparts in Central and South, where the university and its teaching hospitals wielded a powerful influence, North Manchester DHA was not dominated by ‘the powerful presence of teaching professors leaning on everybody’ or ‘self-opinionated and powerful’ consultants (Interview 4: Member of North Manchester DHA). This gave more voice to people from non-medical backgrounds—including, significantly, a local councillor who was heavily involved in both left wing politics and voluntary sector issues and a Community Health Council representative with a background in academic sociology. This very different political balance produced an ethos and context which was far more open to the anti-establishment figure of Judith Gray.

The second source of support came from the Region and its strategy for the rundown and closure of its large mental hospitals (NWRHA, 1985). Although the *Getting to Know* project was clearly independent of any government initiative, it coincided with the Tory administration's final push towards de-institutionalisation. And although North Manchester's proposed new service may not have been quite what the government, nor the regional health authority, had in mind, it fitted the national resettlement agenda perfectly. Because of this the authority were able to apply for regional funding. This took two forms: the first, a 3-year bridging fund grant of £115,000 per annum, financed the initial setting up period, giving the team time to develop the service, prepare the patients and, crucially, to work with the local community prior to discharge. The second was the dowry system, which provided an annual payment of £13,500 (rising to £15,500), guaranteed for life, for each person who was resettled. With the financial backing of the RHA, a project which in normal circumstances would have been dismissed outright by Springfield's finance officers suddenly became not only realisable, but promised a level of resources which most mental health services could only dream of.

Given the project's radical origins and democratic principles, the final source of support was perhaps the most surprising. In September 1986, Edna Robinson, one of a new breed of general managers, took up her post as Sub-unit General Manager, Rehabilitation and Resettlement. Introduced in the context of the Thatcher government's drive for increased efficiency, and with an emphasis on individual accountability, monitoring and cost improvement, general management in the health service was viewed with suspicion and resentment by many professionals (Griffiths, 1983; Ham, 2004; Harrison et al., 1989). For the emergent resettlement team, however, Edna's appointment proved absolutely critical. When she arrived the scheme was already well behind schedule: in the face of uncooperative psychiatrists and a senior nurse manager with her own deployment agenda, only a handful of suitable patients had been identified and there were serious disagreements about the composition and structure of the home support team. Within 6 months the service was up and running, Edna having played a central role not only in overseeing—and indeed expediting—the final stages of the project, but also in galvanising and strengthening support within the hospital. The details of how she achieved this are beyond the remit of this paper. What is significant in this context, however, is the unusual alliance which formed between a mainstream manager, speaking the language of performance management and service models, and a group of radical and/or highly idealistic and committed practitioners—the unexpected but constructive convergence of two apparently contradictory cultures.

## Conclusion: contradictions and convergences; place and space

It was a particular thing in a particular time.

(Interview 6: Member of the *Getting to Know You* Core Planning Group)

The role of Edna Robinson is only one of several contradictions in the story. Another was the way in which such radical ideas were able to take root within the context, firstly of a hugely reactionary organisation and secondly of a strongly right wing government. And third were the cultural contradictions: the unlikely yet productive alliances of radicals and traditionalists, managers and staff, insiders and outsiders; and the convergence of anti-establishment models and mainstream policies and structures.

It is the title of this paper, *Innovation in a Backwater*, which contains the central paradox, however: that ‘the arse-hole of universe’, a marginalised and neglected institution, should become the site of such innovation. And while, as the above quotation captures, this can be partially understood in terms of timing—the fortuitous convergence of local initiatives and national policies, and of key actors brought together within a very particular social, economic and political climate—it is the notion of place which occupies centre stage in any analysis. As Philo and Pickstone (2009) have already highlighted, place encompasses a variety of meanings and dimensions. Echoing Moran and Topp's (2007, p. 4) remarks about ‘the role built space played in narratives of psychiatric progress’, it was, for example, Springfield's appalling physical conditions which served as one of the triggers for service development; while its proximity to—and separation from—the general hospital both reflected and helped shape the institution's ‘unpromising configurations’ of social and political relationships. Within such a marginalised location, ‘the complex intersection between space and surveillance’ (Moran and Topp, 2007, p. 4) played out in unexpected ways, the relative absence of surveillance by Manchester's psychiatric elite creating a space not only for change, but for a change process which was highly unusual in both form and outcome. ‘Space, as Foucault argues, is a metaphor for a site of power which usually constrains but can also liberate’ (Gittins, 1998, p. 5). Sheltered from the constraints of mainstream psychiatry, a space which had more often been associated with oppressive attitudes and backward practices became, however, partially and temporarily, a site of liberation.

## Footnotes

1 The name has been changed to preserve patient confidentiality.

2 Not all would agree that the project constituted a significant innovation. It was never recognised within conventional psychiatric circles and one interviewee likened it to the Emperor's New Clothes—little more than rhetoric. Others, while acknowledging its intrinsic value, have downgraded its wider significance and influence. I would argue, however, that it was important in a number of ways: firstly, it was a genuine, and, during that period, relatively rare attempt to embrace alternative models of mental health care within a statutory setting; secondly, it was absolutely central to the broader cultural changes which Springfield underwent during that period; thirdly, it aroused considerable national interest at the time; and finally, many of the actors involved—a number of whom now have senior positions within mental health—have acknowledged how their experiences with Harpurhey project have strongly influenced their current thinking and practice: ‘We are all who we are because of it partly’ (Interview 6: Member of *Getting to Know You* Core Planning Group)—or, as one interviewee put it ‘some of the sparks… [are] still sparkling’ (Interview 4: Member of North Manchester DHA).

3 Survivors Speak Out, which emerged in 1985, was the first of a series of national networks of service users (Campbell, 2005).

4 R.D. Laing and David Cooper were key figures in what Cooper was the first to term the ‘anti-psychiatry movement’ of the late 1960s and early 1970s. Both psychiatrists were heavily involved in the broader radical politics of the 1960s and in the establishment of a number of therapeutic communities in London, which challenged existing models of and approaches to mental illness. See Crossley (2006) for more details of their work and influence.

5 It was in his inaugural speech to the Annual Conference of the National Association of Mental Health—and with his customary rhetorical flourish—that Powell first introduced his policy of the ‘elimination of by far the greater part of the country's mental hospitals’ which stood ‘isolated, majestic, imperious, brooded over by the gigantic water-tower’ (Jones, 1993, p. 160).

6 NB Although its official name had been changed to the *Department of Psychiatry*, *North Manchester General Hospital* following the 1974 NHS reorganisation it was still known as Springfield Hospital and has consistently been referred to in this way by the people I have interviewed.

7 She has been a leading figure in the Department of Community Medicine in the Medical School of the University of Manchester, where he, an eminent academic dentist, had been Dean. She had moved into the NHS after not being appointed to the professorship in community medicine; he was less radical politically but determined to change North Manchester (Jones and Pickstone, 2008).

8 This first project led to the setting up of Powell St., one of the earliest community mental health centres in the country. Like Harpurhey, it was designed to challenge traditional service models: deliberately located in a terraced house in a back street it was built around the principles of neighbourhood working, community development, user participation and democratic team relationships (Newbigging et al., 1989).

9 The service principles remained a key reference point throughout the team's history: several interviewees have described how the team would refer back to the design principles when disagreements arose; and even when they evolved into a full community mental health team in the early 1990s, they retained their original service principles, including them in their collection of *Key Documents* (Harpurhey and East Manchester Mental Health Service, undated).

10 North Manchester DHA came out of the 1982 NHS reorganisation. Prior to this the district had been part of Manchester Area Health Authority. See Jones and Pickstone (2008) for more details of the reorganisation in Manchester.
